# Women with TSC: Relationship between Clinical, Lung Function and Radiological Features in a Genotyped Population Investigated for Lymphangioleiomyomatosis

**DOI:** 10.1371/journal.pone.0155331

**Published:** 2016-05-12

**Authors:** Fabiano Di Marco, Silvia Terraneo, Gianluca Imeri, Giuseppina Palumbo, Francesca La Briola, Silvia Tresoldi, Angela Volpi, Lorenzo Gualandri, Filippo Ghelma, Rosa Maria Alfano, Emanuele Montanari, Alfredo Gorio, Elena Lesma, Angela Peron, Maria Paola Canevini, Stefano Centanni

**Affiliations:** 1 Respiratory Unit, Ospedale San Paolo, Milan, Italy; 2 Department of Health Science, Università degli Studi di Milano, Milan, Italy; 3 Epilepsy Center, Ospedale San Paolo, Milan, Italy; 4 Diagnostic and Interventional Radiology Unit, Department of Diagnostic Services, Ospedale San Paolo, Milan, Italy; 5 Department of Nephrology, Ospedale San Paolo, Milan, Italy; 6 Dermatologic Clinic, Ospedale San Paolo, Milan, Italy; 7 Disabled Advanced Medical Assistance Unit, Ospedale San Paolo, Milan, Italy; 8 Department of Human Pathology, Cytogenetic and Molecular Pathology, Ospedale San Paolo, Milan, Italy; 9 First Division of Urology, Ospedale San Paolo, Milan, Italy; 10 Laboratories of Pharmacology, Università degli Studi di Milano, Milan, Italy; Imperial College, London, UNITED KINGDOM

## Abstract

The advent of pharmacological therapies for lymphangioleiomyomatosis (LAM) has made early diagnosis important in women with tuberous sclerosis complex (TSC), although the lifelong cumulative radiation exposure caused by chest computer tomography (CT) should not be underestimated. We retrospectively investigated, in a cohort of TSC outpatients of San Paolo Hospital (Milan, Italy) 1) the role of pulmonary function tests (PFTs) for LAM diagnosis, 2) the association between LAM and other features of TSC (e.g. demography, extrapulmonary manifestations, genetic mutations, etc.), and 3) the characteristics of patients with multifocal micronodular pneumocyte hyperplasia (MMPH). Eighty-six women underwent chest CT scan; pulmonary involvement was found in 66 patients (77%; 49% LAM with or without MMPH, and 28% MMPH alone). LAM patients were older, with a higher rate of pneumothorax, presented more frequently with renal and hepatic angiomyolipomas, and tended to have a TSC2 mutation profile. PFTs, assessed in 64% of women unaffected by cognitive impairments, revealed a lower lung diffusion capacity in LAM patients. In multivariate analysis, age, but not PFTs, resulted independently associated with LAM diagnosis. Patients with MMPH alone did not show specific clinical, functional or genetic features. A mild respiratory impairment was most common in LAM-TSC patients: In conclusions, PFTs, even if indicated to assess impairment in lung function, are feasible in a limited number of patients, and are not significantly useful for LAM diagnosis in women with TSC.

## Introduction

Lymphangioleiomyiomatosis (LAM) is a rare progressive cystic lung disease that affects almost exclusively women [1]. LAM can occur sporadically, or can be associated with tuberous sclerosis complex (TSC); a rare disorder with multiorgan involvement effecting the brain, kidneys, heart, liver, skin and eyes and is associated with intellectual disability, epilepsy and autism spectrum disorder [2]. In either form, LAM results from mutations affecting the function of TSC1 or TSC2 genes [3], encoding for hamartin and tuberin, respectively. Such proteins inhibit the mammalian target of the rapamycin (mTOR) signaling pathway, a major regulator of cell size and proliferation [4]. Moreover, TSC patients may develop multifocal micronodular pneumocyte hyperplasia (MMPH), a distinct micronodular epithelial proliferative lesion of the lung, with or without the coexistence of LAM [5]. MMPH is caused by the growth of proliferating epithelial cells into the alveolar walls which is not simply just pneumocyte hyperplasia [5]. Lung function abnormalities in LAM patients include the reduction of both forced expiratory volume in one second (FEV1) and lung diffusion for carbon monoxide (DLCO), which clinically corresponds to a reduction in breathing ability, and hypoxemia when performing physical activity and even at rest [6, 7].

A consensus statement issued by the European Respiratory Society in 2012 defined the diagnostic criteria for LAM [1]. In patients with definite or probable TSC, LAM can be diagnosed on the basis of a characteristic pulmonary high-resolution computed tomography (HRCT) pattern with the presence of more than 10 thin-walled, round and well-defined air-filled cysts with preserved or increased lung volume, and no other significant pulmonary involvement (with the exception of possible features of MMPH) present [1]. In the same document, HRCT scanning is recommended for women with TSC at ages between 18 and 30 years [1]. Previous studies run on women affected by TSC found a LAM prevalence ranging between 26 and 49% [8–13], with an increase of prevalence correlated to age that may reach 81% in subjects aged 40 years or older [10].

Sirolimus and its derivate everolimus are immunosuppressive drugs that affect mTOR function. Both have been demonstrated to be somewhat effective in the treatment of LAM [14–17]. With the advent of such therapies, early diagnosis of LAM has become crucial. However, since the prevalence of clinically significant LAM in TSC patients is low [18–22] and LAM-TSC is a milder disease compared to sporadic LAM [6, 22], the lifelong cumulative radiation exposure risk of serial CT should be taken into account. Cudzilo CJ et al. proposed an age-based approach using limited CT scanning methods in order to facilitate screening and limit radiation exposure [10].

In our study, the evaluation of a possible association between pulmonary and extrapulmonary localization of TSC-related abnormalities was investigated with the objective to assess whether specific extrapulmonary manifestations typical of TSC, or other features of the disease may increase the risk of LAM. The aims were: 1) evaluation of the prevalence of LAM in a large TSC Italian population and usefulness of lung function tests for screening purposes; 2) assessment of the association between LAM-TSC and other features of the disease such as demographic characteristics of patients, the presence of extrapulmonary involvement and the identification of the mutation of gene TSC1 or TSC2; and 3) characterization of patients affected by MMPH alone.

## Methods

### Study design and population

This is a cohort retrospective study involving outpatients affected by TSC, regularly seen at the Tuberous Sclerosis Center of San Paolo Hospital, Milan, Italy, from 2000 to 2014. The diagnosis of TSC was established using international criteria [23]. In our TSC center every systemic manifestation of TSC is evaluated at least yearly by a specialist experienced in TSC diagnosis and management (neurologist, pulmonologist, nephrologist, dermatologist, ophthalmologist, radiologist, and cardiologist) according with international guidelines [23–26]. Pulmonary evaluation with high-resolution lung CT (HRCT) was performed in women [1]: 1) at the age of 18 years for the patients diagnosed with TSC in pediatric age; 2) at the moment of TSC diagnosis in adult patients or during the first evaluation in our center; 3) in case of respiratory symptoms. The analyzed data (demographic, clinical, genetic, pulmonary function tests, and extrapulmonary manifestations) refer to the year of chest CT.

#### Pulmonary involvement

Spirometry, body pletismography and lung diffusion tests (Platinum Elite™ MGC Diagnostic, USA) were performed according to ATS/ERS guidelines [27, 28]. We defined an alteration of pulmonary function test as 1) FEV1/FVC < lower level of normality, 2) reduction of lung diffusion for carbonic monoxide (DLCO) and/or 3) DLCO/alveolar volume (DLCO/VA) < 80% of predicted value using ECCS predicted values [29]. Dyspnea was investigated throughout the Italian version of the modified Medical Resource Council (MRC) scale consisting in five statements regarding perceived breathlessness [30]. The six-minute walk test (6MWT) was performed along a flat, straight, 30 meters walking course supervised by a well-trained researcher according to ATS guidelines [31].

#### Chest CT scans

As previously described, in accordance with the ERS document, the presence of LAM in patients with definite or probable TSC was confirmed in the presence of characteristic lung high-resolution CT patterns [1]. Chest CT examinations were performed without contrast media administration either on a 4-slice multidetector CT (Light Speed QX/i; General Electric Medical System, Milwaukee, WI) between January 2000 and August 2008 or, due to the scan system replacement, with a 64-slice multidetector CT (LightSpeed VCT, General Electric Healthcare, Milwaukee, WI) between September 2008 and December 2014. For both scanners, parameters comprised the following: tube voltage, 100–140 kVp; tube current, 120–400 mAs (with automatic tube current modulation for the 64-slice scanner); gantry rotation time, 0.5 s; reconstruction thickness, 1.25 mm; reconstruction increment, 1.25 mm; acquisition kernel, standard. A beam pitch of 1.5 was used for the 4-slice CT scanner and a pitch of 1 for the 64-slice one. Images were acquired during inspiration and the scan length extended from the lung apices to the adrenal glands.

### Genetic analysis

Qiamp DNA blood mini DNA kit (Qiagen, Germany) was employed to extract DNA from peripheral lymphocytes (Qiagen, Germany). *TSC1* and *TSC2* exons from the genomic DNAs were amplified by means of standard polymerase chain reaction (PCR) and previously described primers [32]. Mutations were detected by submitting the PCR products to denaturing high-performance liquid chromatography (DHPLC) (Transgenomic, Crewe, UK). The products showing variant DHPLC melt profiles were directly sequenced using a BigDye terminator cycle sequencing kit (Applied Biosystems), and the results were analyzed using sequence analysis 3.4.1 software (ABI 3130, Applied Biosystem). The sequencing reactions for identified mutations were repeated. Patients that had negative investigations for DHPLC were evaluated with Multiple Ligation-dependent Probe Amplification test for TSC1 (P124-MRC-Holland) and TSC2 (P046-MRC-Holland). Patients in whom genetic analysis was inconclusive, were classified as having no mutation identified (NMI).

#### Neurological manifestations

Neurological manifestations (cortical tubers, subependymal nodules (SEN) and subependymal giant cell astrocytoma (SEGA)) were evaluated by the use of CT and brain magnetic resonance imaging (MRI). Epilepsy and neurodevelopmental psychiatric/cognitive symptoms were also evaluated. Frequency, age at onset, and characteristics of epilepsy, intellectual disability, sleep disorders and anti-epileptic therapy were reported. Intellectual disability was divided into five grades according to intelligence quotient (IQ): (1) normal IQ with IQ > 85; (2) borderline intellectual functioning (BIF) with IQ from 84 to 71;(3) mild intellectual disability (ID) with IQ from 70 to 55;(4) moderate ID with IQ from 54 to 40, and (5) severe ID with IQ < 40 [33]. As part of their clinical management, patients were evaluated through a psychiatric interview in order to assess possible Axis I and II disorders [33].

#### Abdominal, dermatological and cardiac manifestations

All patients were evaluated at least once with an abdomen CT or MRI [34], and followed-up with ultrasonography (US) in the majority of cases. Abdominal manifestations of TSC include renal angiomyolipomas, renal cysts and renal cell carcinoma and hepatic angiomyolipomas; the data included in our database refer to the closest CT or MRI available, obtained before or after the chest CT-scan. Skin lesions were also clinically evaluated. TSC manifestations include facial angiofibromas, forehead plaques, hypomelanotic macules, shagreen patches and ungual fibroma. Cardiac involvement (rhabdomyoma, electrocardiographic abnormalities) was investigated by electrocardiography and echocardiography.

### Statistics

The results are shown as mean±standard deviation (SD), unless otherwise stated. Lilliefors corrected K-S test was performed before the data analysis in order to examine the distribution of the residuals of the parametric tests. For comparisons between patients, unpaired Student’s t test analysis (test for equal variances was performed), Wilcoxon Mann-Whitney test, or Fisher’s exact test were used, as appropriate. Variables that resulted in p values < 0.15 were used in a multivariate logistic regression model to predict factors that were associated with TSC-LAM diagnosis. The odds ratios (OR) and their 95% confidence intervals were also derived. All tests were two-sided, and p < 0.05 were considered statistically significant. Statistical tests were performed using the Statistical Package for Social Sciences (version 21.0; SPSS, Chicago, IL).

### Ethical considerations

The local ethical committee (Comitato Etico Interaziendale Milano Area A) approved the study. All patients recruited were required to give their signed consent for the collection and analysis of clinical data. Patients with cognitive impairment had consent signed for them by appropriate next of kin.

## Results

### Analysis of the Population and relationship between age and prevalence of LAM in TSC

**Fig 1:**
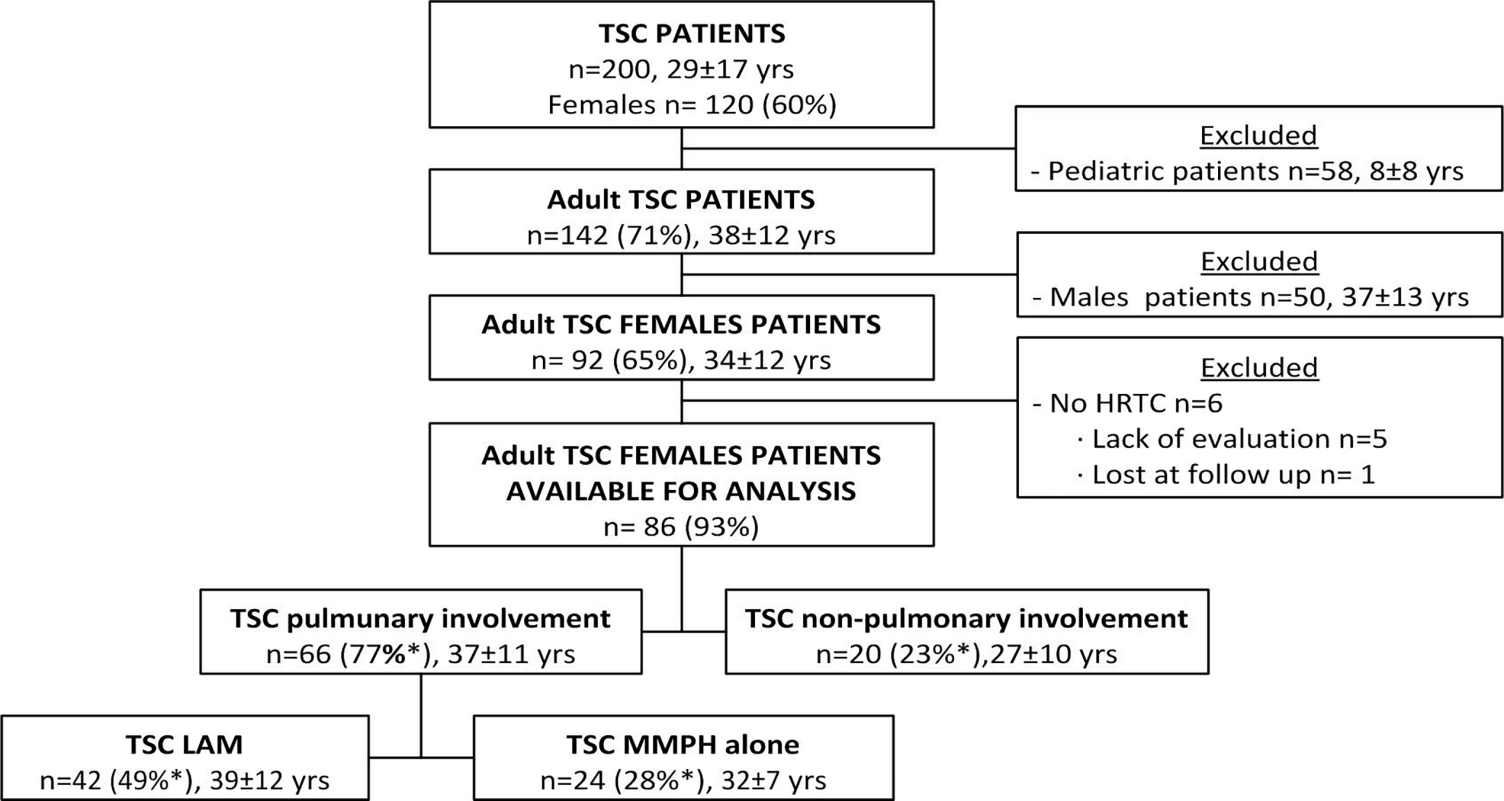
Population in analysis. Age is shown as mean ± standard deviation and is referred to first evaluation in the center. LAM = lymphangioleiomyomatosis; TSC = tuberous sclerosis complex; *percentage referred to all adult TSC females patients in which lung scan was available for evaluation.

Among the 200 patients (80 males, 120 females; mean age 29 years, range 1–71) followed up for TSC at San Paolo Hospital (Milan, Italy) during the period of analysis, 142 were older than 18 years of age (and therefore considered “adult patients”). Ninety-two adult women were evaluated; of them 86 (93%) had chest CT scans (Fig 1). Eighty-two of those scans (95%) were done for screening purposes (requested at the time of the first clinical evaluation); two patients underwent HRTC for pneumothorax, and two others for chylothorax (in these subjects the pulmonary involvement preceded the diagnosis of TSC) during hospitalization. Chest CT scan allowed the following identifications: 66 (77%) adult women had pulmonary involvement with LAM in 42 cases (49%), MMPH in 24 cases (28%), and both LAM and MMPH in 19 cases (22%).

**Fig 2:**
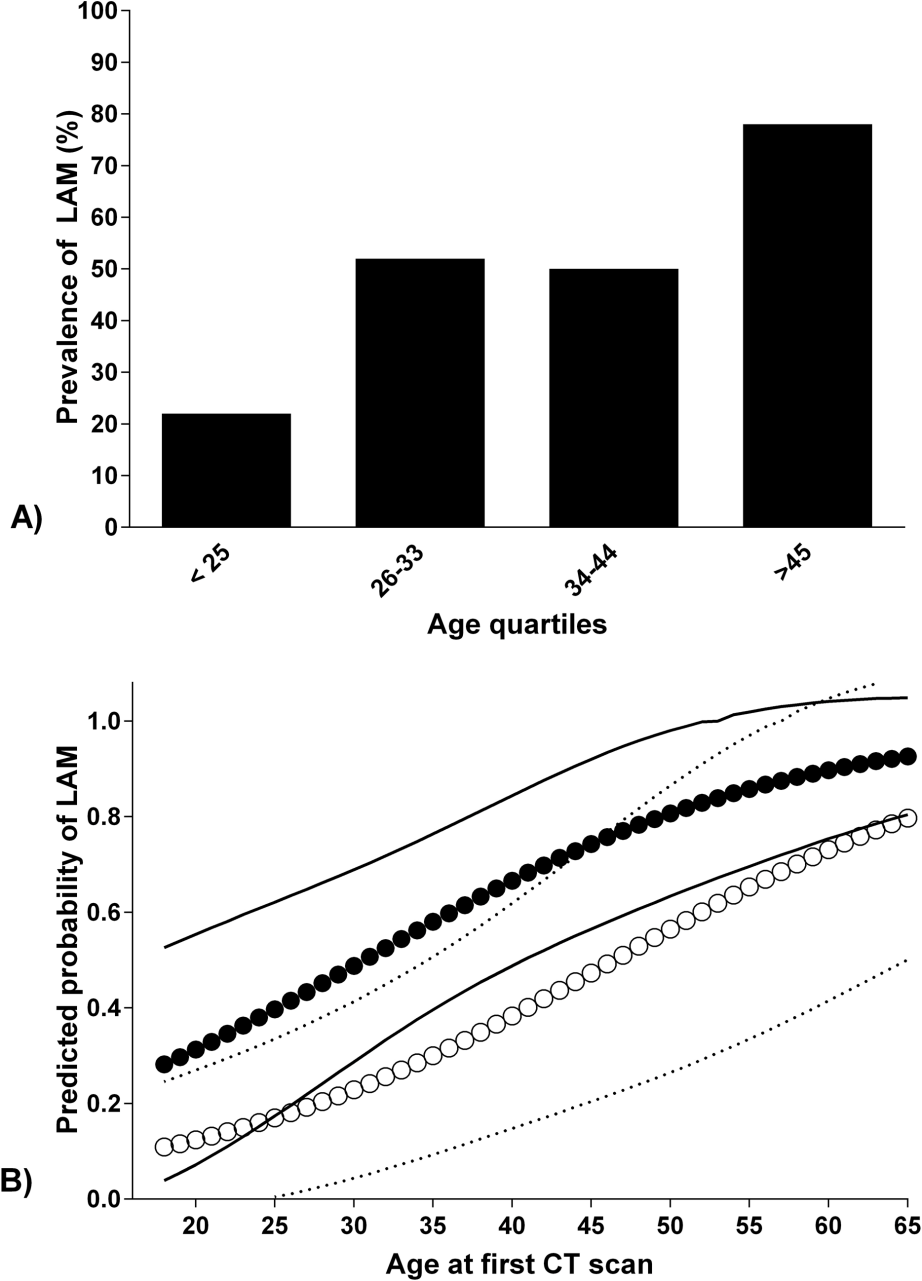
Age-dependent risk of LAM. **(A)** on age quartiles in the overall population (*p = 0*.*004*) **(B)** predicted probability of LAM in relationship to age and 95% CI in patients with and without altered pulmonary function tests. Points along the central logistic curve are individual predicted probabilities. Black points refer to patients with normal pulmonary function tests (PFT), white points refer to patients with altered PFT. The corresponding 95% CI for each point appears on the outer logistic curves. The dotted lines refer to 95% CI of predicted probability for patients with altered PFT while the continuous line refers to IC in patients with normal PFT.

Demographic and clinical features of the population are shown in Table 1. The mean age at first CT evaluation was significantly higher (> 9 years difference) for patients with LAM compared to those without LAM (p< 0.001). LAM prevalence significantly increased across age quartiles (p = 0.005) in the overall population (Fig 2A); TSC2 mutation was found in 50% of the cases. Such percentage changed when the presence of LAM was considered, with a statistically borderline higher prevalence of TSC2 mutation in LAM patients (60 *vs*. 38%, p = 0.070). In the overall population, the most common mutation was “*de novo*” (61%), with the same number of “familial” and “dubious” mutations; in terms of mutations, no significant differences were found in patients with and without LAM (p = 0.282).

**Table 1:** Demographic and clinical characteristics of enrolled patients according with LAM.

	ALL PATIENTS	LAM-TSC	TSC	*P* value
Number (%)	86	42 (49)	44 (51)	
Age at first CT evaluation, yrs	34 ± 12	39 ± 12	30 ± 9	**<0.001**
Genotype^a^				
*TSC1*, n (%)	33 (40)	11 (28)	22 (52)	**0.070**
*TSC2*, n (%)	40 (49)	24 (60)	16 (38)	
No mutation identified (NMI), n (%)	9 (11)	5 (12)	4 (10)	
**Abdominal manifestation**				
Renal angiomyolipomas, n (%)	63 (75)	36 (88)	27 (62)	**0.011**
n <3/n≥ 3, n (%)	9 (25)/ 27 (75)	3 (11)/ 23 (89)	6 (60)/ 4 (40)	**0.006**
bilateral angiomyolipomas, n (%)	32 (80)	23 (88)	9 (64)	0.102
Renal cysts, n (%)	30 (40)	16 (44)	14 (37)	0.636
Hepatic angiomyolipoma, n (%)	29 (35)	22 (54)	7 (17)	**0.002**
**Skin lesions, n (%)**	84 (99)	41 (100)	43 (97)	>0.999
Hypomelanotic macules, n (%)	51 (91)	26 (93)	25 (89)	>0.999
Facial angiofibromas, n (%)	58 (97)	30 (100)	28 (90)	0.492
Fiorhead plaque, n (%)	26 (81)	10 (77)	16 (84)	0.666
Shagreen patches, n (%)	13 (41)	7 (50)	6 (33)	0.473
Ungual fibromas, n (%)	30 (77)	18 (95)	12 (60)	**0.020**
**Neurological manifestation**				
Epilepsy, n (%)	54 (64)	22 (54)	32 (72)	0.076
Cortical tubers, n (%)	78 (92)	37 (88)	41 (95)	0.265
Subependymal nodules, n (%)	58 (76)	29 (78)	29 (74)	0.790
SEGA, n (%)	13 (17)	8 (22)	5 (13)	0.591
Sleep disorders, n (%)	58 (89)	26 (87)	32 (91)	0.695
Intellectual disability, n (%)	35 (44)	19 (47)	16 (40)	0.652
Borderline, n (%)	5 (6)	4 (10)	1 (2)	0.586
Level 1, n (%)	10 (12)	5 (12)	5 (12)	
Level 2, n (%)	8 (10)	3 (7)	5 (12)	
Level 3, n (%)	12 (15)	7 (17)	5 (12)	
**Ocular manifestation**				
Fundus oculi abnormalities, n (%)	32 (73)	15 (68)	17 (77)	0.736
Retinic amartomas, n (%)	6 (46)	2 (29)	4 (67)	0.286
**Cardiac manifestation**				
Cardiac rhabdomyomas, n (%)	12 (17)	4 (11)	8 (22)	0.343

Results are shown as mean± standard deviation unless otherwise stated. SEGA: subependymal giant cell astrocytoma; IQR: interquartile range; SD: standard deviation. p < 0.050 in bold.

^a^Results of genetic analysis were available for 82 TSC patients.

### Pulmonary involvement, symptoms and respiratory function

Respiratory function tests were successfully carried out in 55 patients (64%), due to the high prevalence of intellectual disability and/or behavioral problems (Table 2); namely, 14 patients were not co-operative, in 7 cases spirometry was not acceptable due to glottis closure, 5 had variable effort with early termination of forced expiration and 5 patients were not able to perform reproducible tests. LAM patients showed lower DLCO and DLCO/VA (both referred to predicted values); the difference in terms of obstruction (i.e. FEV1/FVC ratio under lower limit of normality, LLN) between LAM and TSC-LAM resulted in borderline statistical significance (p = 0.080). Impairment in lung function tests is more common in TSC-LAM patients than in TSC patients without LAM (p = 0.055). As shown in Fig 2B, patients with an altered lung function showed a higher percentage of LAM with age. Five TSC-LAM patients had a history of recurrent pneumothorax. Patients with or without LAM did not differ in dyspnoea and oxygen desaturation during 6MWT. We also evaluated the usefulness of pulmonary function tests in the subgroup of 16 women with “respiratory impairment” (i.e. dyspnoea, pneumothorax, or chylothorax). The prevalence of LAM in this subgroup did not differ significantly from asymptomatic patients (26 *vs*. 11%, p = 0.068), such as the results of pulmonary function tests, with the only exception of a borderline reduction of DLCO (i.e. <80%) in patients with symptoms (38 vs. 14%, p = 0.050).

**Table 2:** Lung function, pulmonary manifestations and symptoms of patients according with LAM.

		ALL PATIENTS	LAM-TSC	TSC	P value
**Lung function**^**a**^					
FEV1 (% pred), median (IQR)		95 (85–106)	95 (85–106)	94 (86–108)	0.736
FVC (% pred)		99 ± 17	100 ± 19	97 ± 15	0.607
FEV1/FVC ratio, median (IQR)		101 (96–104)	99 (94–103)	102 (97–104)	0.166
FEV1/FVC ratio < LLN, n (%)		6 (11)	5 (21)	1 (3)	0.080
DLCO (% pred)		81 ± 18	74 ± 22	86 ± 14	**0.029**
VA (% pred)		96 ± 15	93 ±16	98 ± 14	0.229
DLCO/VA (% pred)		81 ± 18	74 ± 20	87 ± 15	**0.007**
DLCO/VA < 80% pred, n (%)		25 (46)	16 (64)	9 (31)	**0.028**
VR (% pred)		131 ± 59	140 ± 65	127 ± 56	0.502
TGV (% pred)		116 ±34	123 ± 38	112 ± 31	0.358
Alteration of PFT, n (%)		32 (58)	19 (73)	13 (44)	**0.055**
**Lung involvement**^**b**^					
Smoke history (current/ex), n (%)		8 (9)/ 1 (1)	6 (14)/ 1 (3)	2 (4)/ 0 (0)	0.166
Pneumothorax, n (%)		5 (6)	5 (12)	0 (0)	**0.024**
Chylothorax		3 (3)	3 (7)	0 (0)	0.112
MMPH, n (%)		43 (50)	19 (45)	24 (55)	0.518
Dyspnea, n (%)		14 (17)	9 (23)	5 (11)	0.241
	mMRC = 1, n (%)	2 (14)	1 (11)	1 (20)	0.486
	mMRC >1, n (%)	12 (86)	8 (89)	4 (80)	
SpO2 < 90% during 6mWT, n (%)		11 (14)	4 (10)	7 (18)	0.518

Results are shown as mean± standard deviation unless otherwise stated. PFT: pulmonary function test (alteration: FEV1/FVC < LLN, and/or DLCO < 80%, and/or DLCO/VA < 80%); IQR: interquartile range; FEV1: forced expiratory volume in one second; FVC: forced expiratory volume; LLN: lower limit of normality; DLCO: diffusion capacity for CO; VA: alveolar volume; TLC: total lung capacity; RV: residual volume; TGV: thoracic gas volume; MMPH: multifocal micronodular pneumocyte hyperplasia; mMRC: Modified Medical Research Council Dyspnea Scale, %pred: % of predicted value; p < 0.050 in bold.

^a^Data and percentage referred to 55 patients who performed lung function tests

^b^Data and percentage referred to 86 patients with CT scan.

### Extrathoracic involvement and MMPH patients

Renal (multiple and bilateral), and hepatic angiomyolipomas were significantly more frequent in patients with LAM, compared to those without LAM (p = 0.011, and p = 0.002, respectively). In addition, women with LAM less frequently had a history of epilepsy than patients without LAM (p = 0.076) (Table 1).

Twenty-five patients with MMPH alone (i.e. without LAM) have been compared with 24 patients with no pulmonary involvement. Patients having MMPH alone did not differ from the other TSC patients without any lung manifestation in terms of clinical features, neuropsychiatric symptoms, genetic characteristics, and lung function tests (Table 3).

**Table 3:** Demographic, pulmonary, clinical characteristic and genetic analysis of TSC patients according with MMPH.

	MMPH-TSC	TSC	*P value*
Number	24	20	
Age at first CT evaluation, (yrs)	32 ± 7	27 ± 10	0.074
Dyspnea, n (%)	4 (83)	4 (17)	0.362
Smoke history (current or past), n (%)	1 (4)	1 (5)	>0.999
SO2 < 90% during 6mWT, n (%)	6 (29)	1 (5)	0.095
**Respiratory function**			
FEV1 (% pred)	96 ± 13	98 ± 8	0.550
FEV1/FVC < LLN, n (%)	1 (5)	0 (0)	>0.999
FVC (% pred)	98 ± 16	97 ±13	0.806
DLCO (% pred)	84 ± 13	90 ±15	0.297
VA (% pred)	100 ±15	96 ±14	0.562
DLCO/VA (% pred)	86 ±13	89 ± 17	0.697
RV (% pred)	125 ±63	128 ±31	0.881
**Genotype**			
*TSC1*, n (%)	14 (58)	8 (44)	0.355
*TSC2*, n (%)	9 (37)	7 (39)	
No mutation identified (NMI), n (%)	1 (4)	3 (17)	
**Abdominal manifestations**			
Renal angiomyolipomas, n (%)	12 (50)	15 (79)	0.064
Hepatic angiomyolipoma, n (%)	3 (14)	4 (21)	0.760
**Skin involvement**^**a**^, n (%)	24 (100)	19 (95)	0.455
**Neurological manifestations**			
Epilepsy, n (%)	18 (75)	14 (70)	0.746
Brain tubers, n (%)	23 (100)	18 (90)	0.210
Intellectual disability, n (%)	7 (33)	9 (47)	0.520
**Ocular manifestations**			
Retinic hamartoma, n (%)	4 (100)	0 (0)	0.067
Cardiac hamartoma, n (%)	3 (15)	5 (31)	0.422

Results are shown as mean± standard deviation unless otherwise stated. IQR: interquartile range; FEV1: forced expiratory volume in one second; FVC: forced expiratory volume; LLN: lower limit of normality; DLCO: diffusion capacity for CO; VA: alveolar volume; TLC: total lung capacity; RV: residual volume; TGV: thoracic gas volume; yrs: years; %pred: % of predicted value.

^a^Any skin manifestation of TSC.

### Predictors of TSC-LAM

Pulmonary function test alterations alone yielded a sensitivity of 45% and specificity of 70% for LAM diagnosis by using CT scan as gold standard, with a positive and negative predicted value of 59% and 57% respectively. A multivariate model was used to estimate the odds for LAM diagnosis. The only element found in the univariate analysis that was independently associated with LAM diagnosis in our TSC group was the age at first CT evaluation, with a higher risk of LAM in older women (Table 4). Our analysis failed to demonstrate alterations of PFT as independently associated with LAM in TSC patients (p = 0.245).

**Table 4:** Multivariate Analysis and ODDS RATIO for LAM risk in overall population.

	UNIVARIATE			MULTIVARIATE		
Variable	OR	95% CI	p value	OR	95% CI	p value
Haepatic AML	4.26	1.39–13.09	0.011	-	-	-
Renal AML	2.26	1.02–5.00	0.430	-	-	-
Altered PFT	3.34	1.07–10.38	0.037	-	-	-
TSC1/TSC2	1.52	0.79–2.95	0.207	-	-	-
Age	1.08	1.03–1.13	0.001	1.083	1.014–1.156	**0.018**

*AML*: *angiomyolipoma; PFT*: *pulmonary function test; TSC1/2/NMI*: *mutation of TSC1 TSC2 genes/ no mutation identified; Age*: *referred to age at first CT evaluation; p < 0*.*050 in bold*.

## Discussion

The main findings of this study, conducted on a large cohort of Italian TSC patients, are the following: 1) LAM and MMPH have a prevalence of 49% and 28% respectively; 2) on average, women with LAM are older, develop renal and hepatic angiomyolipomas more frequently, show a higher rate of pneumothoraces, and have more mutations on the TSC2 gene; 3) Impairment in lung function tests, feasible in patients not affected by major cognitive deficit (64%), is more common in LAM patients; 4) older age is independently associated with LAM whereas multivariate analysis failed to demonstrate pulmonary function test alterations as an independent risk factor for LAM diagnosis; 5) patients with MMPH alone do not show a specific clinical, functional or genetic profile.

To the best of our knowledge, this is the first study that describes the clinical characteristics of a large Italian population of patients with LAM associated with TSC and that investigates the possible role of respiratory function test in detecting pulmonary involvement.

Our data, in line with previous studies [8–13], indicate that in TSC patients there is an age-related LAM prevalence, a higher frequency of TSC2 mutations (statistically borderline in our study) [11, 35, 36], and more frequent occurrence of renal [10, 11, 22] and hepatic angiomyolipomas [37]. The crucial importance of “time” in the manifestation of lung lesions is probably due to the pathogenesis of LAM-TSC, which is consistent with the Knudson “two-hit” tumor suppressor gene mechanism [38]. Moreover, in line with previous data, our results support the evidence that patients with TSC1 mutations have, on average, milder disease in comparison with patients with TSC2 mutations [13, 39].

We encountered two major difficulties in the use of pulmonary function tests in TSC patients: firstly, 36% of TSC women who underwent chest CT failed to correctly perform spirometry and other pulmonary function tests due to TSC-related intellectual disability. This could potentially be aided by the use of other techniques that require a lower level of co-operation, such as using forced oscillation technique (FOT), a simple, noninvasive method which requires minimal patient technical ability, currently used in both children and adults. The second problem was the low sensitivity (<50%) of PFTs for LAM screening in TSC women. This may be due to numerous issues, e.g. TSC patients usually present with a mild form of LAM, LAM is the initial presenting symptom of TSC only occasionally, and the decline in lung function is typically very gradual in patients with LAM-TSC, with only a minority of patients becoming symptomatic during follow-up [40–42]. However, an interesting study conducted by Taveira-DaSilva AM et al. showed that some young LAM-TSC patients (mean age 26±3 years) can rapidly progress from minimal to severe lung disease [22]. The real question is whether it is important to diagnose LAM in asymptomatic patients, which on average present with very mild lung disease. Screening for LAM in TSC patients should take into consideration potential benefits and risks. Potential benefits of earlier LAM diagnosis include the possibility to inform women about the risk of a pneumothorax, pregnancy, the use of contraceptives, and lifestyle choices, such as scuba diving or smoking, as well as the opportunity to start mTOR inhibitor therapy, even if this choice is currently limited to patients with lung function declining rapidly or respiratory symptoms, chylous pleural effusion or ascites [43]. On the other hand, the risk of carcinogenic ionizing radiation exposure has to be taken into account, as well as the possible anxiety due the diagnosis, and lifestyle limitations (e.g. the risk of pneumothorax and air travel) not supported by a strong level of evidence (e.g. the risk of a life-threatening pneumothorax associated with air travel is minor) [44].

Our study confirms that, so far, HRCT is the only available tool for LAM screening in TSC patients. Multivariate analysis failed to identify a parameter that is independently associated with the presence of LAM, with the only exception of age. To conclude, the need of LAM diagnosis by chest HRCT scan in asymptomatic TSC women with normal lung function should be weighed in each individual circumstance with consideration of the pros and cons.

The significant percentage of TSC patients showing MMPH (50%) correlates halfway between previous reports [11] and a recent work by Muzykewicz et al. [45] that found a nodular lesion prevalence of 57% in TSC patients. We did not find a significant correlation between the presence of MMPH and LAM since the rate of MMPH was the same in patients with and without LAM (p = 0.518). Moreover, even if not confirmed by statistically significant results, MMPH alone seems to be more common in older patients (5 years of difference compared to TSC patients without any lung involvement, p = 0.074). However, the presence of MMPH itself does not affect pulmonary function, with pulmonary function tests on average normal in those patients and comparable with TSC women without evidence of lung disease. Thus, the evidence of MMPH at HRCT should be considered as clinically negligible, with the exception, in our experience, of occasional atypical radiological findings which require follow-up in order to exclude other diagnosis (e.g. in situ adenocarcinoma).

A number of potential limits of the present study deserve discussion. First, due to the retrospective nature of our work, we did not determine any biomarker, such as vascular endothelial growth factor-D (VEGF-D) at time of first HRTC, which demonstrated a potential clinical utility in reaching a diagnosis of LAM [46]. The correlation between higher values of VEGF-D and a reduced lung function has been found in some but not all studies [46–50]. However, no study has specifically investigated the potential role of the combination of pulmonary function tests and biomarkers for the diagnosis of LAM, reason why this approach is worth of investigation. Secondly, the understanding of TSC and LAM has significantly changed in the period of our data collection. Even if this is not a limit for pulmonary function tests, since spirometry and DLCO are unchanged for decades, we cannot rule out a possible bias for changes in the evaluation of extrapulmonary manifestation of the disease in the 14 years of data collection. Third, as suggested by many international documents, CT-scan evaluation was limited to women, since the presence of cysts in men is anecdotal. The reason being we cannot rule out some LAM-like lesions that are present in men with TSC, as previously reported [51]. We also did not analyze the data of abdominal lymphangioleiomyomas or of lymphadenopathy since scarcity in the number of patients. Finally the, power of our multivariate analysis is low, due to the limited number of patients with all the parameters available for analysis.

## Conclusions

Impairment in lung function tests is common in LAM, but pulmonary function testing, needed to evaluate the level of lung impairment, does not prove to be a useful tool for detecting LAM in TSC women in clinical practice. Using more sensitive tests which require a lower level of co-operation could assist, if available. However, the weak correlation between lung function impairment, "anatomical" lung cysts and symptoms limits the utility of lung function testing for LAM in patients with TSC. The use of low dose CT methods are suggested to limit the lifelong cumulative radiation exposure.
